# Clinical characteristics of second primary cancer in colorectal cancer patients: the impact of colorectal cancer or other second cancer occurring first

**DOI:** 10.1186/1477-7819-12-73

**Published:** 2014-03-28

**Authors:** Li-Chu Sun, Yi-Yun Tai, Su-Mien Liao, Tang-Yu Lin, Ying-Ling Shih, Se-Fen Chang, Ching-Wen Huang, Hon-Man Chan, Che-Jen Huang, Jaw-Yuan Wang

**Affiliations:** 1 Department of Nutrition Support Team, Kaohsiung Medical University Hospital, Kaohsiung 807, Taiwan; 2 Department of Nursing, Kaohsiung Medical University Hospital, Kaohsiung 807, Taiwan; 3 Faculty of Post-baccalaureate Medicine, Kaohsiung Medical University, Kaohsiung 807, Taiwan; 4 Department of Nursing, Shu-Zen College of Medicine and Management, Kaohsiung 821, Taiwan; 5 Division of Gastrointestinal and General Surgery, Department of Surgery, Kaohsiung Medical University Hospital, Kaohsiung 807, Taiwan; 6 Department of Surgery, Kaohsiung Municipal Hsiao-Kang Hospital, Kaohsiung Medical University, Kaohsiung, Taiwan; 7 Department of Surgery, Faculty of Medicine, College of Medicine, Kaohsiung Medical University, Kaohsiung 807, Taiwan; 8 Graduate Institute of Clinical Medicine, College of Medicine, Kaohsiung Medical University, Kaohsiung 807, Taiwan; 9 Graduate Institute of Medicine, College of Medicine, Kaohsiung Medical University, Kaohsiung 807, Taiwan; 10 Cancer Center, Department of Surgery, Kaohsiung Medical University Hospital, Kaohsiung 807, Taiwan

**Keywords:** Colorectal cancer, Second primary cancer, Prognosis

## Abstract

**Background:**

Due to improvements in early detection, treatment, and supportive care, the number of colorectal cancer (CRC) survivors is increasing; therefore, careful attention should always be paid to the second primary cancer (SPC) in treating these CRC patients. The present study attempts to determine the correlation and clinical aspects of CRC to other cancers in patients suffering from SPC involving CRC.

**Methods:**

From January 2002 and June 2010, 1,679 cancer cases, CRC was accompanied by SPC in 89 patients (5.3%), including 16 (18%) synchronous and 73 (82%) metachronous SPC patients. These patients were subsequently classified into two groups: the first group had CRC diagnosed first as CRC first (CRCF); and the second group had another type of cancer diagnosed before the diagnosis of CRC as other cancer first (OCF). Of these 73 patients, 22 (30.1%) were in the group of CRCF, whereas 51 (69.9%) were in the group of OCF. Patients’ clinicopathological characteristics and clinical outcomes were analyzed and compared between the two groups.

**Results:**

There was a significant difference in the sites of cancers between the two groups: 14 (27.5%) patients in the OCF group had gastric cancer, compared to one (4.5%) patient in the CRCF group (*P* = 0.026). Although there was no difference of hepatitis B virus (HBV) or hepatitis C virus (HCV) carriers between the OCF and CRCF groups (*P* = 0.165), there were six (27.3%) CRC patients with hepatocellular carcinoma (HCC) in the CRCF group, which was significantly higher than the two (3.9%) patients in the OCF group (*P* = 0.003). Furthermore, the cancer-specific survival rate of the CRCF patient group was significantly higher than that of the OCF patient group (*P* = 0.036).

**Conclusions:**

In this retrospective analysis, gastric cancer patients compared to other secondary cancers were at a higher risk of developing subsequent CRC as SPC; alternatively, patients with CRC were at a higher risk of developing HCC as SPC subsequently, no matter whether patients were HBV or HCV carriers. Therefore, careful attention should always be paid to the possibility of secondary CRC to construct effective surveillance when treating cancer patients.

## Background

Colorectal cancer (CRC) is the second leading cause of cancer-related mortality in Europe and the USA, and there are approximately 300,000 new cases and 200,000 deaths due to CRC in these areas annually [1,2]. It has been reported that the incidence of CRC in economically transitioning countries continues to rise and the incidence of CRC in economically developed countries has stabilized or is declining [3]. In Taiwan, CRC was the third leading cause of cancer death and the death rate was 20.2 per 100,000 in 2010 (http://www.mohw.gov.tw/cht/DOS/Statistic.aspx?f_list_no=312&fod_list_no=2622; accessed in Auguest 2012). There was a 24.69% increase in the CRC-related death rate in 2010 compared with that in 2002 and a 64.23% increase compared with that in 1996 [1]. In Taiwan, cancer is reported as the first leading cause of death and the death rate was 177.40 per 100,000 in 2010, over eight times the European and USA average. There was a 16.02% increase in the cancer death rate in 2010 compared with that in 2002 and a 36.04% increase compared with that in 1996 (http://www.mohw.gov.tw/cht/DOS/Statistic.aspx?f_list_no=312&fod_list_no=2622; accessed in Auguest 2012). Cancer patients are at high risk of developing a second cancer after the treatment of the initial cancer [1,4,5]. Due to improvements in early detection, supportive care, and multimodality treatments, the number of CRC survivors has increased in recent decades, and continuing careful attention should always be paid to the second primary cancer (SPC) in treating these CRC patients.

In recent years, reports have documented that the present percentage of SPC in the total number of cancers ranges from 0.73% to 11.70% [6] and the trend is increasing [5,7]. Compared with the general population, patients with CRC are at a higher risk of developing a SPC [8]. Thus, it is important to be aware of the clinical characteristics of SPC in CRC patients for early diagnosis and effective treatment. Several analytical studies have found interesting associations between the first tumor site and the subsequent tumors; this could be useful for prevention [9-11]. The initial characteristics of CRC as well as possible risk factors for subsequent SPC are also crucial for clinicians [12,13]. However, no relevant information regarding the differences between the sequences of CRC or SPC occurrence is available up to the present time.

In this retrospective analysis, CRC patients with at least two primary malignancies were analyzed with regard to differences of clinicopathological features and survival (CRC first or other malignancy first). We tried to identify potential risk factors and clinical outcomes which could possibly evaluate, describe, and provide appropriate postoperative surveillance strategies for CRC patients and furthermore, identify early SPC.

## Methods

### Patients

This retrospective cohort study included 1,679 consecutive patients with histologically proven CRC who received surgical treatment at the Department of Surgery at Kaohsiung Medical University Hospital, Kaohsiung,Taiwan, from January 2002 to December 2010. The inclusion criteria were that each tumor had to have a definite histological picture of primary CRC and the probability that one was a metastatic lesion from the other organ must be excluded [11]. Of 1,679 cancer cases, CRC was accompanied by SPC in 89 patients (5.3%), including 16 (18%) synchronous and 73 (82%) metachronous SPC. SPC detected within 6 months after the initial cancers were regarded as synchronous cancers, and the others were categorized as metachronous cancers [10]. Therefore, 16 synchronous cancer patients were excluded for the subsequent analysis. The patients were classified into two groups: one group had CRC diagnosed first as CRC first (CRCF); and the other group had another type of cancer diagnosed before the diagnosis of CRC as other cancer first (OCF).

Patients’ clinicopathological characteristics were analyzed. Available variables included: age of onset, gender, tumor location, histological type, TNM classification of malignant tumors (TNM) defined according to the criteria of the American Joint Commission on Cancer/Union for International Cancer Control (AJCC/UICC) [14], vascular invasion, perineural invasion, preoperative serum level of albumin, preoperative serum level of carcinoembryonic antigen (CEA), comorbidity of cardiac disease, administration of chemotherapy, and body mass index (BMI). Preoperative serum levels of albumin and CEA were checked within 1 week before operation. The normal cut-off values of serum albumin and CEA were defined as 3.5 gm/dL and 5 ng/mL, respectively. We reviewed all patients’ charts to identify the presence of secondary cancers. Patient clinical outcomes and survival status were regularly followed up until their last visit or death, or if the patient was lost to follow-up. This present study is a retrospective cohort study by analysis from medical records, it dose not need patients' ICF according to Taiwan local IRB regulation after approval of the Institutional Review Board of the Kaohsiung Medical University Hospital. (KMUH-IRB-990361).

### Statistical analysis

All data were statistically analyzed using IBM SPSS Statistics, version 17.0 (IBM Corporation, Armonk, NY, USA). For the univariate statistical analysis, chi-square test and student *t*-test were used where applicable for categorical and continuous variables, respectively. Cancer-specific and overall survival rates were calculated by the Kaplan − Meier method, and the differences in survival rates were analyzed by the log-rank test. A *P* value of less than 0.05 was considered to be statistically significant.

## Results

The clinical and pathological data regarding CRC patients diagnosed with SPC are summarized in Table 1. There were 73 (4.3%) patients diagnosed with metachronous SPC. Sixty-eight (93.2%) patients had double cancers and five (6.8%) patients suffered from triple cancers. Of these 73 patients, 22 (30.1%) were in the group of CRCF, whereas 51 (69.9%) were in OCF. There were also no significant differences in gender, tumor size, tumor location, histological type, AJCC/UICC cancer stage, vascular invasion, perineural invasion, BMI, and comorbidity of pulmonary disease and renal disease. Meanwhile, the percentages of patients receiving adjuvant chemotherapy were not significantly different in the two groups (*P* = 0.206; Table 2). The mean age at the time of diagnosis of the first cancer was 64.0 years in the CRCF group and 58.0 years in OCF patients. Likewise, the mean age at the time of the second cancer between the two groups was not significantly different (67.0 ± 12.0 versus 66.9 ± 13.6). Between the first and second cancer there is a time lag of approximately 2.9 years in the CRCF group and 8.7 years in the OCF group, which was significantly different (*P* = 0.002; Table 2).

**Table 1:** Clinicopathological characteristics of 73 second primary cancer (SPC) patients

**Parameters**	**Case number**	**Percentage (%)**
Gender		
Male/Female	34/39	46.6/53.4
Age (years)		
≥65/<65	42/31	57.5/42.5
HBV and HCV carrier		
Yes/No	13/60	17.8/82.2
BMI (kg/m^2^)		
<22/≥22	34/39	46.6/53.4
Albumin (gm/dL)		
<3.5/≥3.5	32/41	43.8/56.2
Tumor size (cm)		
≥5/<5	29/44	39.7/60.3
Tumor location		
Right colon/Left colon/Rectum^a^	18/35/20	24.7/47.9/27.4
TNM stage		
I/II/III/IV	12/26/24/11	16.4/35.6/32.9/15.1
Vascular invasion		
Yes/No	24/49	32.8/67.2
Perineural invasion		
Yes/No	22/51	30.1/69.7
Histology type		
PD/MD/WD	8/57/8	11.0/78.0/11.0
Depth		
T1/T2/T3/T4	6/7/53/7	8.2/9.6/72.6/9.6
Lymph node		
N0/N1/N2	42/22/9	57.5/30.2/12.4
Pre-operation CEA (ng/mL)		
≥5/<5	39/34	53.4/46.6
Chemotherapy		
Yes/No	42/31	57.5/42.5
Diabetes mellitus		
Yes/No	15/58	20.5/79.5
Cardiac disease		
Yes/No	17/56	23.3/76.7

^a^Right colon: cecum, ascending colon, and transverse colon; left colon: descending colon and sigmoid colon. BMI, body mass index; CEA, carcinoembryonic antigen; HBV, hepatitis B virus; HCV, hepatitis C virus; MD, moderately differentiated; PD, poorly differentiated; SPC, second primary cancer; TNM, TNM classification of malignant tumors; WD, well differentiated.

**Table 2:** Clinicopathological characteristics of 73 second primary cancer (SPC) patients by other cancer first (OCF) and colorectal cancer first (CRCF)

**Parameters**	**OCF (%)**	**CRCF (%)**	***P *** **value**
	**N = 51 (69.9)**	**N = 22 (30.1)**	
Gender			
Male/Female	23 (45.1)/28 (54.9)	11 (50.0)/11 (50.0)	0.700
Age (years)			
<65/≥65	21 (41.2)/30 (58.8)	10 (45.5)/12 (54.5)	0.734
Interval diagnosis time (years)	8.7 ± 8.32	2.9 ± 1.98	0.002
HBV and HCV carrier			
Yes/No	7 (13.7)/44 (86.3)	6 (27.3)/16 (72.7)	0.165
BMI (kg/m^2^)			
<22/≥22	24 (47)/27 (53)	10 (45.5)/12 (54.5)	0.916
Albumin (gm/dL)			
<3.5/≥3.5	24 (47)/27 (53)	8 (36.3)/14 (63.7)	0.390
Tumor size (cm)			
≥5/<5	23 (45.1)/28 (54.9)	6 (27.3)/16 (72.7)	0.136
Tumor location^a^			0.158
Right colon	15 (29.4)	3 (13.6)	
Left colon	25 (49.0)	10 (45.5)	
Rectum	11 (21.6)	9 (40.9)	
TNM stage			
III + IV/I + II	26 (51)/25 (49)	9 (40.9)/13 (59.1)	0.429
Vascular invasion			
Yes/No	16 (31.4)/35 (68.6)	8 (36.4)/14 (63.6)	0.632
Perineurial invasion			
Yes/No	18 (35.3)/33 (64.7)	4 (18.2)/18 (81.8)	0.159
Histology type			
PD/WD + MD	6 (11.8)/45 (88.2)	2 (9.1)/20 (90.9)	0.864
Depth			
T3 + T4/T1 + T2	43 (84.3)/8 (15.7)	17 (77.3)/5 (22.7)	0.635
Lymph Node			
N1 + N2/N0	23 (43.1)/28 (54.9)	8 (36.4)/14 (63.6)	0.488
Pre-operation CEA (ng/mL)			
≥5/<5	25 (49)/26 (51)	14 (63.6)/8 (36.4)	0.226
Chemotherapy			
Yes/No	27 (52.9)/24 (47.1)	15 (68.2)/7 (31.8)	0.206
Diabetes mellitus			
Yes/No	11 (21.6)/40 (78.4)	4 (18.2)/18 (81.8)	0.742
Cardiac disease			
Yes/No	5 (9.8)/46 (90.2)	4 (18.2)/18 (81.8)	0.318

^a^Right colon: cecum, ascending colon, and transverse colon; left colon: descending colon and sigmoid colon. BMI, body mass index; CEA, carcinoembryonic antigen; CRCF, colorectal cancer first; HBV, hepatitis B virus; HCV, hepatitis C virus; MD, moderately differentiated; OCF, other cancer first; PD, poorly differentiated; SPC, second primary cancer; TNM, TNM tumor node metastasis; WD, well differentiated.

Table 3 shows the sites of the SPCs. In the 15 sites of SPC, gastric cancer, breast cancer, bladder cancer, and hepatocellular carcinoma (HCC) were most frequently detected, followed by cancers in the lung. The most frequent site of cancers in the group of OCF was gastric cancer, and the most frequent site of cancers in the group of CRCF were HCC and ovarian cancer. There was a significant difference in the sites of cancers in these two groups: 14 (27.5%) patients in the OCF group had gastric cancer compared to one (4.5%) patient in the CRCF group (*P* = 0.026). The comparison stage data of 15 gastric cancer patients and eight HCC patients between CRCF and OCF was not significantly different (*P* = 0.232 versus *P* = 0.587; Tables 4 and 5). Although there was no difference of hepatitis B virus (HBV) or hepatitis C virus (HCV) carriers between the OCF and CRCF (*P* = 0.165), a measure of six (27.3%) of 22 CRC patients with HCC as SPC in the CRCF group was significantly higher than two (3.9%) of 51 patients in the OCF group (*P* = 0.003). There was a significant difference in the sites of cancers in these two groups: no patients in the OCF group had ovarian cancer compared to two (9.1%) patients in the CRCF group (*P* = 0.029).

**Table 3:** Site distribution of second primary cancers (SPCs) in patients with other cancer first (OCF) and colorectal cancer first (CRCF)

**Parameters**	**OCF (%)**	**CRCF (%)**	***P *** **value**
	**(N = 51)**	**(N = 22)**	
Gastric cancer	14 (27.5)	1 (4.5)	0.026
Hepatocellular carcinoma (HCC)	2 (3.9)	6 (27.3)	0.003
Breast cancer	10 (19.6)	2 (9.1)	0.266
Prostate cancer	2 (3.9)	0 (0)	0.346
Cervical cancer	3 (5.9)	0 (0)	0.245
Ovarian cancer	0 (0)	2 (9.1)	0.029
Bladder cancer	4 (7.8)	4 (18.2)	0.194
Lung cancer	2 (3.9)	2 (9.1)	0.373
Thyroid cancer	5 (9.8)	0 (0)	0.128
Nasal pharyngeal cancer	4 (7.8)	0 (0)	0.177
Lymphoma	2 (3.9)	1 (4.5)	0.902
Oral cancer	3 (5.9)	1 (4.5)	0.818
Gallbladder cancer	0 (0)	1 (4.5)	0.125
Thymus cancer	0 (0)	1 (4.5)	0.125
Brain cancer	0 (0)	1 (4.5)	0.125
HBV and HCV carrier	7 (13.7)	6 (27.3)	0.165

CRCF, colorectal cancer first; HBV, hepatitis B virus; HCV, hepatitis C virus; OCF, other cancer first; SPC, second primary cancer.

**Table 4:** Comparison stage of gastric cancer between other cancer first (OCF) and colorectal cancer first (CRCF)

**Parameters**	**OCF (%)**	**CRCF (%)**	***P *** **value**
	**N = 14 (93.3)**	**N = 1 (6.7)**	
TNM stage I	2 (14.3)	1 (100)	0.232
TNM stage II	4 (28.6)	0 (0)	
TNM stage III	3 (21.4)	0 (0)	
TNM stage IV	5 (35.7)	0 (0)	

CRCF, colorectal cancer first; OCF, other cancer first; TNM, TNM classification of malignant tumors.

**Table 5:** Comparison stage of hepatocellular carcinoma (HCC) between other cancer first (OCF) and colorectal cancer first (CRCF)

**Parameters**	**OCF (%)**	**CRCF (%)**	***P *** **value**
	**N = 2 (25)**	**N = 6 (75)**	
TNM stage I	0 (0)	1 (100)	0.587
TNM stage II	1 (50)	0 (0)	
TNM stage III	1 (50)	0 (0)	
TNM stage IV	0 (0)	0 (0)	

CRCF, colorectal cancer first; HCC, hepatocellular carcinoma; OCF, other cancer first; TNM, TNM classification of malignant tumors.

**Figure 1:**
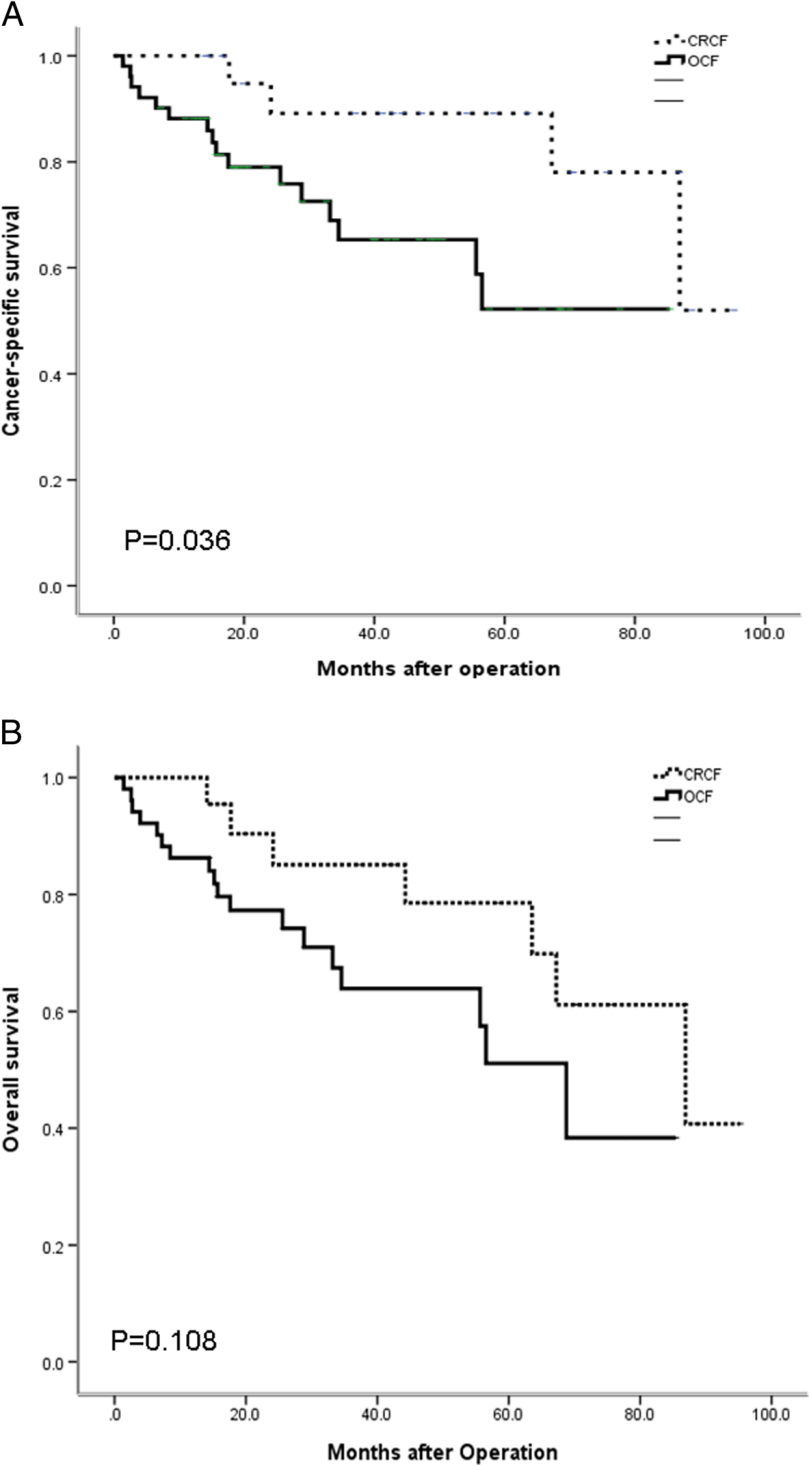
**Cumulative survival rates of second primary cancer (SPC) in colorectal cancer (CRC) patients. (A)** Cumulative cancer-specific survival rates of SPC in CRC patients. Better survival was observed in the colorectal cancer first (CRCF) group (*P* = 0.036). **(B)** Cumulative overall survival rates of SPC in CRC patients. No significant differences were observed between the CRCF and other cancer first (OCF) group (*P* = 0.108). CRC, colorectal cancer; CRCF, colorectal cancer first; OCF, other cancer first; SPC, second primary cancer.

Regarding survival analysis, the cancer-specific survival of CRCF patients was significantly higher than that of the OCF patient group (*P* = 0.036; Figure 1A), but overall survival of CRCF patients was not significantly different between the two groups (*P* = 0.108; Figure 1B).

## Discussion

Cancer-bearing patients are assumed to have an increased incidence of developing cancer of other organs [15]. In this current investigation, we identified that 5.3% of CRC patients were diagnosed with SPC, which was consistent with previous investigations (range between 0.73% and 11.70%) [6], and the trend has been increasing in recent reports [5,7]. The most frequent sites of cancers preceding or following CRC were as follows: gastric cancer, breast cancer, bladder cancer, and HCC. In Western studies, however, different combinations of common sites of multiple cancers are reported, most likely due to the low incidence of gastric cancer in Western populations.

Of the 51 cases in the OCF group, the stomach was the most frequent site before developing CRC as SPC. Ueno et al. indicated that when colon cancer was combined with other cancers, stomach cancer (1.4%) was the most frequently encountered neoplasm, followed by breast cancer (0.4%); and when rectal cancer was combined with other cancers, stomach cancer (0.6%) was also the most frequently encountered neoplasm, but this was followed by lung cancer (0.5%) [4]. Consequently, they suggested close follow-up to ascertain the possibility of developing secondary CRC among gastric cancer patients postoperatively. Our results are consistent with this study, since the most frequent SPC after gastric cancer was CRC. Therefore, colonoscopic examination should be arranged for the possibility of CRC in treating gastric cancer patients if they have hematochezia or ileus, in order to detect synchronous or metachronous CRC [13]. On the other hand, the liver was the most common site in CRC patients who developed SPC in the present study; however, gastric cancer remains the most frequent SPC in CRC patients from a Japanese study [4]. Despite there being no difference of HBV or HCV carriers between the OCF and CRCF, the incidence of HCC as SPC in the CRCF group was significantly higher than that in the OCF group. Patients with CRC were at a high risk of developing HCC as SPC, no matter whether the patients were HBV or HCV carriers. Follow-up strategies such as abdominal echo or computed tomography (CT) are also needed for these CRC patients to detect primary or metastatic liver lesions.

Likewise, in this study, the incidence of lung cancer together with CRC was only 0.5%, which is close to the result of the Japanese study [4]. Another study from Taiwan observed that the most common SPC in CRC patients was the liver, followed by the stomach [16]. In our study, HCC is ranked as the third leading SPC. However, only 1.26% of patients developed SPCs in another study from northern Taiwan [16]. A significantly higher proportion of Dukes’ B patients developed SPC than that of patients of other stages [16]. In our study, nine (0.53%) of TNM stage II patients developed SPC, which was not significantly different from other stages.

Birgisson et al. reveals that inclusion of SPC affects the results of cancer-specific survival rates in patients with CRC [17]. SPC generates a significant number of events during follow-up of patients with CRC, which causes worse cancer-specific survival rates when SPC is included as an event in the calculations. Almost half as many SPC cases were observed as endpoints as death from the same cancer or non-cancer-related deaths [17]. For women diagnosed with both breast cancer and CRC, the cumulative risk of death at 5 years following the second cancer diagnosis is three times more likely to be due to CRC than to breast cancer [18]. In our study, the cancer-specific survival rate of CRCF patients was significantly higher than that of the OCF patients. The study by Tsai et al. suggests that perineural invasion may be a significant factor for postoperative early relapse of colon cancer and significantly lower overall survival rates in CRC patients [19]. Despite the cancer-specific survival rate being higher in the CRCF group, the overall survival was not significantly different between the CRCF and OCF groups in our study, and this might result from the cause of death being non-cancer-related death (>60% after 8 years of follow-up). The event that occurred most often in patients treated with curative intention was non-cancer-related death [18].

We are left, finally, with several caveats: 1) there are few (if any) prospective controlled trials of screening strategies among patients who are at risk for second malignancies that can guide us with objective, evidence-based information about appropriate screening and management for patients with primary malignancies; 2) there is very little validated evidence that an increased screening frequency will improve outcomes among patients who develop second malignancies; and 3) the optimal screening modalities and strategies for patients who are at risk for second malignancies remain to be defined for most tumor sites. Additional investigative efforts in the future should strive to address these limitations in our current knowledge.

## Conclusion

In summary, SPC in CRC remains a difficult issue for clinicians no matter whether in CRCF or OCF. In addition to a regular follow-up program, for the variety of different sites of SPC, clinicians must pay more attention to determining the potential lesions especially for the accompanying high incidence of malignancies.

## Abbreviations

AJCC: American Joint Commission on Cancer; BMI: Body mass index; CEA: Carcinoembryonic antigen; CRC: Colorectal cancer; CRCF: Colorectal cancer first; CT: Computed tomography; HBV: Hepatitis B virus; HCC: Hepatocellular carcinoma; HCV: Hepatitis C virus; OCF: Other cancer first; SPC: Second primary cancer; TNM: TNM classification of malignant tumors; UICC: Union for International Cancer Control.

## Competing interests

The authors declare that they have no competing interests.

## Authors’ contributions

LCS and YYT analyzed the data and wrote the manuscript. SML, TYL, YLS, SFC, CWH, HMC and CJH made substantial contributions to data acquisition, statistical analyses and data interpretation, and helped in manuscript preparation. JYW participated in study design and coordination. All authors read and approved the final manuscript.
